# Semaphorin7A Promotion of Tumoral Growth and Metastasis in Human Oral Cancer by Regulation of G1 Cell Cycle and Matrix Metalloproteases: Possible Contribution to Tumoral Angiogenesis

**DOI:** 10.1371/journal.pone.0137923

**Published:** 2015-09-17

**Authors:** Tomoaki Saito, Atsushi Kasamatsu, Katsunori Ogawara, Isao Miyamoto, Kengo Saito, Manabu Iyoda, Takane Suzuki, Yosuke Endo-Sakamoto, Masashi Shiiba, Hideki Tanzawa, Katsuhiro Uzawa

**Affiliations:** 1 Department of Oral Science, Graduate School of Medicine, Chiba University, 1-8-1 Inohana, Chuo-ku, Chiba, 260–8670, Japan; 2 Department of Dentistry and Oral-Maxillofacial Surgery, Chiba University Hospital, 1-8-1 Inohana, Chuo-ku, Chiba, 260–8670, Japan; 3 Division of Oral Surgery and Oral Implant Center, Funabashi Central Hospital, 6-13-10 Kaijin, Funabashi, Chiba, 273–8556, Japan; 4 Department of Molecular Virology, Graduate School of Medicine, Chiba University, 1-8-1 Inohana, Chuo-ku, Chiba, 260–8670, Japan; 5 Division of Oral Surgery, Chiba Rosai Hospital, 2–16 Tatsumidaihigashi, Ichihara, Chiba, 290–0003, Japan; 6 Department of Environmental Health Science, Graduate School of Medicine, Chiba University, 1-8-1 Inohana, Chuo-ku, Chiba, 260–8670, Japan; 7 Department of Clinical Oncology, Graduate School of Medicine, Chiba University, 1-8-1 Inohana, Chuo-ku, Chiba, 260–8670, Japan; China Medical University, TAIWAN

## Abstract

**Background:**

Semaphorins (SEMAs) consist of a large family of secreted and membrane-anchored proteins that are important in neuronal pathfinding and axon guidance in selected areas of the developing nervous system. Of them, SEMA7A has been reported to have a chemotactic activity in neurogenesis and to be an immunomodulator; however, little is known about the relevance of SEMA7A in the behaviors of oral squamous cell carcinoma (OSCC).

**Methods:**

We evaluated SEMA7A expression in OSCC-derived cell lines and primary OSCC samples using quantitative reverse transcriptase-polymerase chain reaction, immunoblotting, and semiquantitative immunohistochemistry (sq-IHC). In addition, SEMA7A knockdown cells (shSEMA7A cells) were used for functional experiments, including cellular proliferation, invasiveness, and migration assays. We also analyzed the clinical correlation between SEMA7A status and clinical behaviors in patients with OSCC.

**Results:**

SEMA7A mRNA and protein were up-regulated significantly (P<0.05) in OSCC-derived cell lines compared with human normal oral keratinocytes. The shSEMA7A cells showed decreased cellular growth by cell-cycle arrest at the G1 phase, resulting from up-regulation of cyclin-dependent kinase inhibitors (p21^Cip1^ and p27^Kip1^) and down-regulation of cyclins (cyclin D1, cyclin E) and cyclin-dependent kinases (CDK2, CDK4, and CDK6); and decreased invasiveness and migration activities by reduced secretion of matrix metalloproteases (MMPs) (MMP-2, proMMP-2, pro-MMP-9), and expression of membrane type 1- MMP (MT1-MMP). We also found inactivation of the extracellular regulated kinase 1/2 and AKT pathways, an upstream molecule of cell-cycle arrest at the G1 phase, and reduced secretion of MMPs in shSEMA7A cells. sq-IHC showed that SEMA7A expression in the primary OSCCs was significantly (P = 0.001) greater than that in normal counterparts and was correlated with primary tumoral size (P = 0.0254) and regional lymph node metastasis (P = 0.0002).

**Conclusion:**

Our data provide evidence for an essential role of SEMA7A in tumoral growth and metastasis in OSCC and indicated that SEMA7A may play a potential diagnostic/therapeutic target for use in patients with OSCC.

## Introduction

Semaphorins (SEMAs), secreted and membrane-associated proteins, provide environmental cues to mediate diverse developmental processes including neuronal cellular migration, axon guidance, vasculogenesis, branching morphogenesis, and cardiac organogenesis [1]. SEMA abnormalities have been implicated in the pathogenesis of neurologic disorders, such as Alzheimer’s disease and motor neuron degeneration. SEMAs also are expressed in the immune response systems, including B cells, T cells, natural killer cells, and macrophages, and have been implicated in regulation of organogenesis, angiogenesis, apoptosis, and neoplasia [2].

SEMA1-8 are characterized by the presence of a conserved large SEMA domain (∼500 amino acids) at the N-terminal domain and differentiated by their C-terminus [3]. SEMA1 and SEMA2 are found in invertebrates, SEMA3-7 are found in vertebrates, and SEMA8 is found in viruses [4]. SEMA4-7 exist primarily as membrane-bound forms, whereas SEMA3 is secreted as a soluble molecule. Diffusible SEMAs can elicit autocrine/paracrine signaling, while membrane-bound family members can mediate short-range juxtacrine signals.

Although cancer cells typically express abnormal levels of SEMAs, the role of SEMA7A in cancer progression is largely unknown. SEMA7A, a novel transmembrane glycosylphosphatidylinisotol-anchored protein, was first identified in the immune system in myeloid and lymphoid lineage cells [5–7] and functions through β-integrins in multiple systems [8]. We present the results of a comprehensive analysis of molecular/cellular subtypes of SEMA7A in oral squamous cell carcinoma (OSCC) that are linked functionally and contribute clinically to tumoral progression and prognosis in OSCCs.

## Materials and Methods

### Ethical statement

The Ethical Committee of the Graduate School of Medicine, Chiba University (approval number, 236) approved the study protocol, which was performed in accordance with the tenets of the Declaration of Helsinki. All patients provided written informed consent.

### OSCC-derived cell lines and tissue specimens

Human OSCC-derived cell lines (HSC-2, HSC-3, HSC-4, Sa3, Ca9-22, SAS, KOSC-2, Ho-1-u-1, and Ho-1-N-1) were obtained from the Human Science Research Resources Bank (Osaka, Japan) or the RIKEN BioResource Center (Ibaraki, Japan) through the National Bio-Resource Project of the Ministry of Education, Culture, Sports, Science and Technology in Japan. Short tandem repeat profiles confirmed cellular identity. Primary cultured human normal oral keratinocytes (HNOKs) were obtained from healthy oral mucosa epithelium specimens collected from young patients at Chiba University Hospital. Three independent HNOKs were primary cultured and maintained in oral keratinocyte medium (ScienCell Research Laboratories, Carlsbad, CA, USA) comprised of 5 ml of oral keratinocyte growth supplement (ScienCell Research Laboratories) and 5 ml of penicillin/streptomycin solution (ScienCell Research Laboratories) [9–12]. All OSCC-derived cells were grown in Dulbecco’s modified Eagle medium (DMEM) (Sigma-Aldrich, St. Louis, MO, USA) supplemented with 10% fetal bovine serum (FBS) (Sigma-Aldrich) and 50 units/ml penicillin and streptomycin (Sigma-Aldrich).

One-hundred and fifty primary OSCC samples and patient-matched normal epithelium were obtained during surgeries performed at Chiba University Hospital. The resected tissues were fixed in 20% buffered formaldehyde solution for pathologic diagnosis and immunohistochemistry (IHC) staining. We performed histopathological diagnosis of each OSCC sample according to the World Health Organization criteria at the Department of Pathology of Chiba University Hospital [13]. The clinicopathological stages were determined based on the TNM classification of the International Union against Cancer [14].

### mRNA expression analysis

Total RNA was isolated using Trizol Reagent (Invitrogen, Carlsbad, CA, USA), according to the manufacturer’s instructions. cDNA was generated by using ReverTra Ace qPCR RT Master Mix (Toyobo Life Science, Osaka, Japan) according to the manufacturers’ instructions. Real-time quantitative reverse transcriptase-polymerase chain reaction (qRT-PCR) was performed in a 20-μl reaction volume using the Thunderbird SYBR qPCR Mix (Toyobo) on the LightCycler 480 apparatus (Roche Diagnostics, Mannheim, Germany), according to the manufacturer’s protocol. The general amplification conditions were performed as described previously [15–18]. Primers were designed using Primer 3Plus (on-line free software, http://primer3plus.com/), which specifies the most suitable set. The primer sequences used for qRT-PCR were: *SEMA7A*, forward, 5′-TGTGTATTCCCTCGGTGACA-3′; reverse, 5′-GAGTGGAACAATGGCGTCTT-3′; and *glyceraldehyde-3-phosphate dehydrogenase* (*GAPDH*), forward, 5′-AACATCATCCCTGCCTCTACTGG-3′; reverse, 5′-TTGAAGTCAGAGGAGACCACTG-3′; and *MMP2*, forward, 5′-CCCCAAAACGGACAAAGAG-3′; reverse, 5′-CTTCAGCACAAACAGGTTGC-3′; and *MMP9*, forward, 5′-GAACCAATCTCACCGACAGG-3′; reverse, 5′-GCCACCCGAGTGTAACCATA-3′; and *membrane type 1- MMP* (*MT1-MMP*), forward, 5′-GCCTTGGACTGTCAGGAATG-3′; reverse, 5′-AGGGGTCACTGGAATGCTC-3′. The transcript amount for SEMA7A was estimated from the respective standard curves and normalized to the *GAPDH* transcript amount determined in corresponding samples.

### Immunoblotting analysis

The cells were washed three times with cold phosphate buffered saline (PBS) and gently and briefly centrifuged. The cellular pellets were incubated at 4°C for 30 minutes in a lysis buffer (7 M urea, 2 M thiourea, 4% w/v CHAPS, and 10 mM Tris, pH 7.4) with a proteinase inhibitor cocktail (Roche Diagnostics). The total protein concentration was measured using a dye-binding method based on the Bradford assay with Bio-Rad Protein Assay Dye Reagent Concentrate (Bio-Rad Laboratories, Hercules, CA, USA).

Protein extracts were electrophoresed on 4–12% Bis-Tris gel and transferred to nitrocellulose membranes (Invitrogen) and blocked for 1 hour at room temperature with Blocking One (Nacalai Tesque, Inc., Kyoto, Japan). The membranes were washed three times with 0.1% Tween-20 in Tris-buffered saline (TBS-T) and incubated with affinity-purified mouse anti-SEMA7A monoclonal antibody (Santa Cruz Biotechnology, Santa Cruz, CA, USA), rabbit anti-GAPDH polyclonal antibody (Santa Cruz Biotechnology), rabbit anti-cyclin D1 polyclonal antibody (Santa Cruz Biotechnology), rabbit anti-cyclin E1 polyclonal antibody (Santa Cruz Biotechnology), rabbit anti-p21^Cip1^ polyclonal antibody (Santa Cruz Biotechnology), rabbit anti-AKT polyclonal antibody (Santa Cruz Biotechnology), rabbit anti- phosphorylated-AKT (pAKT) polyclonal antibody (Santa Cruz Biotechnology), rabbit anti- membrane type-1 matrix metalloproteinase (MT1-MMP) monoclonal antibody (Cell Signaling Technology, Danvers, MA, USA), rabbit anti-extracellular signal-regulated kinase (ERK) 1/2 (Cell Signaling Technology), rabbit anti-phosphorylated-ERK1/2 (pERK1/2) (Cell Signaling Technology), rabbit anti-p27^Kip1^ polyclonal antibody (Cell Signaling Technology), rabbit anti-cyclin-dependent kinase (CDK) 2 monoclonal antibody (Cell Signaling Technology), rabbit anti-CDK4 monoclonal antibody (Cell Signaling Technology), and rabbit anti-CDK6 monoclonal antibody (Cell Signaling Technology) overnight at 4°C. The membrane was washed with TBS-T and incubated with horseradish peroxidase-conjugated anti-rabbit or anti-mouse IgG as a secondary antibody (Promega, Madison, WI, USA), for 1 hour at room temperature. Finally, the membranes were detected using Super-Signal West Pico Chemiluminescent substrate (Thermo Fisher Scientific, Rockford, IL, USA), and immunoblotting was visualized by exposing the membranes to ChemiDoc XRS Plus system (Bio-Rad Laboratories). The signal intensities were quantitated using the Image Lab system (Bio-Rad Laboratories). Densitometric SEMA7A protein data were normalized to GAPDH protein levels.

### Transfection with shRNA plasmid

OSCC-derived cells (SAS and KOSC-2) were transfected with SEMA7A shRNA (shSEMA7A) or control shRNA (shMock) vectors (Santa Cruz Biotechnology) with Lipofectamine 3000 and Plus Reagents (Invitrogen), according to the manufacturer’s instructions. After transfection, the cells were isolated by the culture medium containing 1 μg/ml puromycin (Invitrogen). Several weeks after transfection, a small colony was viable. The cell colonies were picked, transferred to six-well plates, and expand gradually to 10-cm dishes. To assess the efficiency of SEMA7A knockdown, we performed qRT-PCR and immunoblotting.

### Cellular growth

To evaluate the effect of SEMA7A knockdown on cellular proliferation, we analyzed cellular growth in shSEMA7A and shMock cells. These cells were seeded in 6-cm dishes at a density of 1 × 10^4^ viable cells. At the indicated time points, the cells were trypsinized and counted in triplicate using a hemocytometer.

### Invasiveness assay

To evaluate the effect of SEMA7A knockdown on invasiveness, a total of 2.5×10^5^ cells resuspended in the serum-free medium were seeded on Matrigel^®^-coated Transwell^®^ inserts (8 μm pores) (Becton-Dickinson, Franklin Lakes, NJ, USA). In the lower chamber, 2 ml of DMEM with 10% FBS was added as a chemoattractant. After the cells were incubated for 48 hours at 37°C, the insert was washed with PBS, and the cells on the top surface of the insert were removed with cotton swabs. Cells adhering to the lower surface of the membrane were fixed with methanol and stained with crystal violet. The number of cells that invaded through the pores in five random fields was counted using a light microscope at ×100 magnification.

### Migration assay

The cells were seeded in six-well plates with 10% FBS/DMEM until a confluent monolayer formed. Using a micropipette tip, one wound was created in the middle of each plate. We incubated plates at 37°C at 5% carbon dioxide with free-serum medium. The results were visualized by measuring the wound area that was free of cells using the Lenaraf220b software (http://www.vector.co.jp/soft/dl/win95/art/se312811.html). The mean value was calculated from data obtained from six wells.

### Cell-cycle analysis

The transmutants were treated with 200 ng/ml nocodazole (Sigma-Aldrich) for 16 hours to synchronize cells at the G2/M transition [19–21]. Sixteen hours after treatment with nocodazole, the cells were harvested, washed with PBS, and probed with the CycleTEST Plus DNA reagent kit (Becton-Dickinson). Flow cytometric determination of the DNA content was analyzed using the BD Accuri^TM^ C6 Flow Cytometer (Becton-Dickinson).

### Zymography

To detect the secreted MMPs from shSEMA7A and shMock cells, we carried out a gelatin zymography assay (Primary Cell, Sapporo, Japan), according to the manufacturer’s instructions with minor modifications. The conditioned media were mixed with an SDS sample buffer and separated on gelatin gels. After washing, the gels were incubated for 32 hours at 37°C in the enzymatic reaction buffer. The gels then were stained with Coomassie brilliant blue and destained in a methanol/acetic acid solution with gentle agitation on a shaking plate. The MMPs were identified by the presence of clear bands in a background with uniform staining.

### Cycloheximide (CHX) treatment

CHX, a common reagent for inhibition of protein synthesis, has been reported to inactive the MMPs functions. Since several studies have showed that the Ki of CHX ranges from 1 to 50 μg/ml [22–27], we used CHX (WAKO, Osaka, Japan) at a concentration of 1 μg/ml for 48 hours. After treatment, immunoblotting analysis and zymography were performed.

### Transient up-regulation in SEMA7A knockdown cells

Recent study has been reported that TGF-β_1_ (R&D Systems, Minneapolis, MN, USA) increases the expression level of SEMA7A through a SMAD2/3-independent pathway [28]. Since, we have treated shSEMA cells with TGF-β_1_ at a concentration of 1 ng/mL for 12 hours. After treatment, mRNA expression and immunoblotting analyses were performed.

### Semiquantitative IHC

Semiquantitative IHC (sq-IHC) of the 4-μm sections of paraffin-embedded OSCC clinical specimens was performed. Briefly, after paraffinization, hydration, activation of antigen, hydrogen peroxide quenching and blocking, the clinical sections were incubated with mouse anti-SEMA7A monoclonal antibody (Santa Cruz Biotechnology) at 4°C in a moist chamber overnight. Upon incubation with the primary antibody, the specimens were washed three times with PBS and treated with Envision reagent (DAKO, Carpinteria, CA, USA) followed by color development in 3,3’-diaminobenzidine tetrahydrochloride (DAKO). The slides then were counterstained lightly with hematoxylin, dehydrated with ethanol, cleaned with xylene, and mounted. To quantify the status of the SEMA7A protein expression in clinical samples, we used the sq-IHC scoring systems described previously [19, 29–33]. The mean percentages of positive tumoral cells were determined in at least three random fields in each section, and the intensity of the SEMA7A-immunoreaction was scored as follows: 0+, none; 1+, weak; 2+, moderate; and 3+, intense. The staining intensity and the cellular numbers were multiplied to produce a SEMA7A sq-IHC score. To determine the cutoff points of the SEMA7A sq-IHC scores, we analyzed the sq-IHC scores of 150 patients using receiver operating characteristic (ROC) curves and the Youden index. Cases with a score over 69.5 were defined as SEMA7A-positive. Two independent pathologists from Chiba University Hospital, neither of whom had knowledge of the patients’ clinical status, made these judgments. To calculate the 5-year survival rate, we surveyed each patient’s life and month of death.

### Statistical analysis

To compare SEMA7A expression levels, statistical significance was evaluated using the Mann-Whitney U test. Relationships between SEMA7A sq-IHC staining scores and clinicopathological profiles were evaluated using the χ^2^ test, Fisher’s exact test, Student’s t-test, and Mann-Whitney U test. The 5-year survival rate was evaluated using the log-rank test. P<0.05 was considered statistically significant. The data are expressed as the mean ± the standard error of the mean (SEM).

## Results

### Evaluation of SEMA7A expression in OSCC-derived cell lines

**Fig 1:**
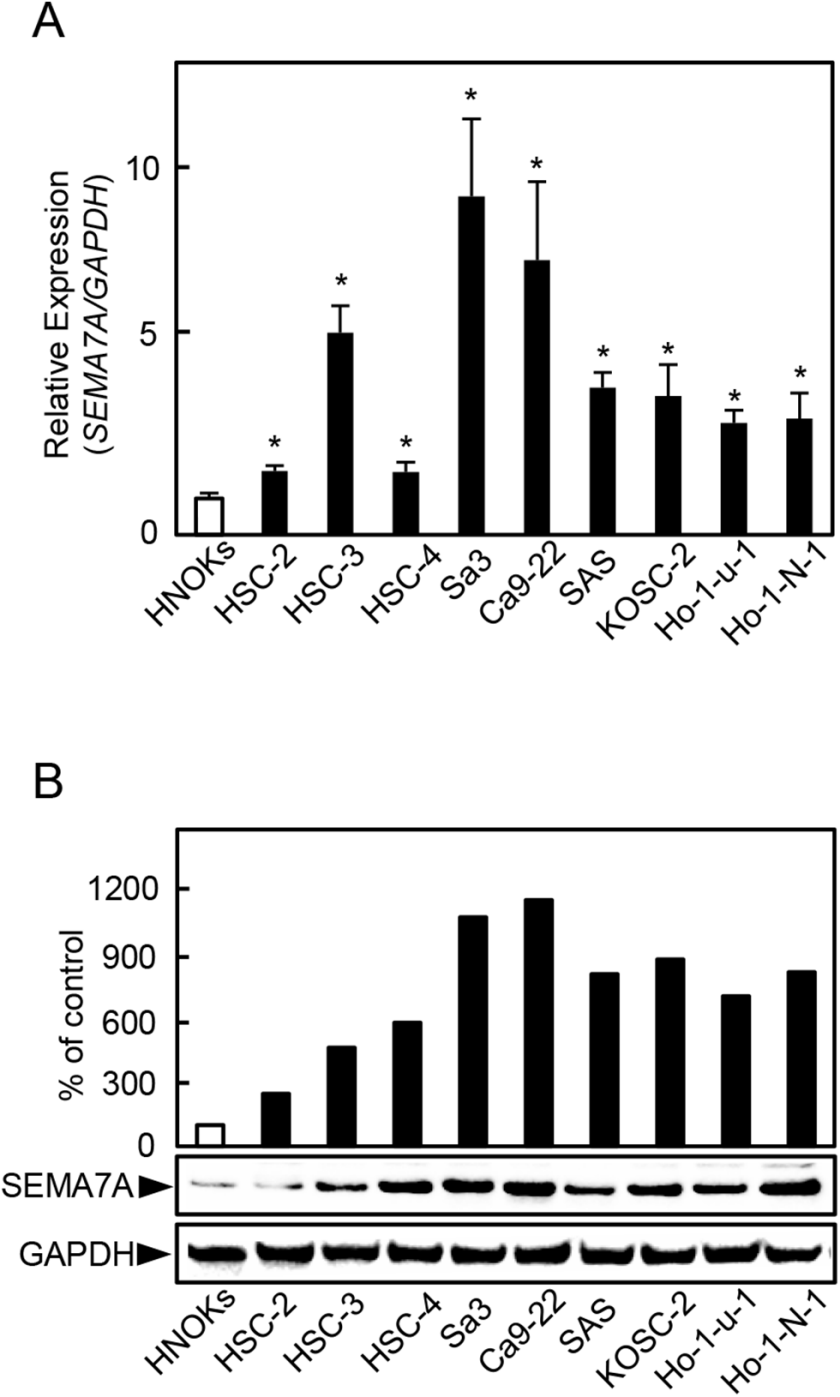
Evaluation of SEMA7A expression in OSCC-derived cell lines. (**A**) Quantification of *SEMA7A* mRNA expression in OSCC-derived cell lines by qRT-PCR analysis. Significant (^✽^P<0.05, Student’s t-test) up-regulation of *SEMA7A* mRNA is seen in seven OSCC-derived cell lines compared with the HNOKs. Data are expressed as the mean ± SEM of triplicate results. (**B**) Immunoblotting analysis of SEMA7A protein in OSCC-derived cell lines and HNOKs. SEMA7A protein expression is up-regulated in OSCC-derived cell lines compared with that in the HNOKs. Densitometric SEMA7A protein data are normalized to the GAPDH protein levels. The values are expressed as a percentage of the HNOKs.

To investigate the expression status of SEMA7A, we performed qRT-PCR and immunoblotting analyses using nine OSCC-derived cell lines (HSC-2, HSC-3, HSC-4, Sa3, Ca9-22, SAS, KOSC-2, Ho-1-u-1, and Ho-1-N-1) and HNOKs. *SEMA7A* mRNA was up-regulated significantly (P<0.05) in all OSCC-derived cell lines compared with the HNOKs (Fig 1A). We also performed immunoblotting analysis to investigate the SEMA7A protein expression in the OSCC-derived cell lines and the HNOKs (Fig 1B). A significant increase in SEMA7A protein expression was seen in all OSCC-derived cell lines compared with the HNOKs.

### Establishment of SEMA7A knockdown cells

**Fig 2:**
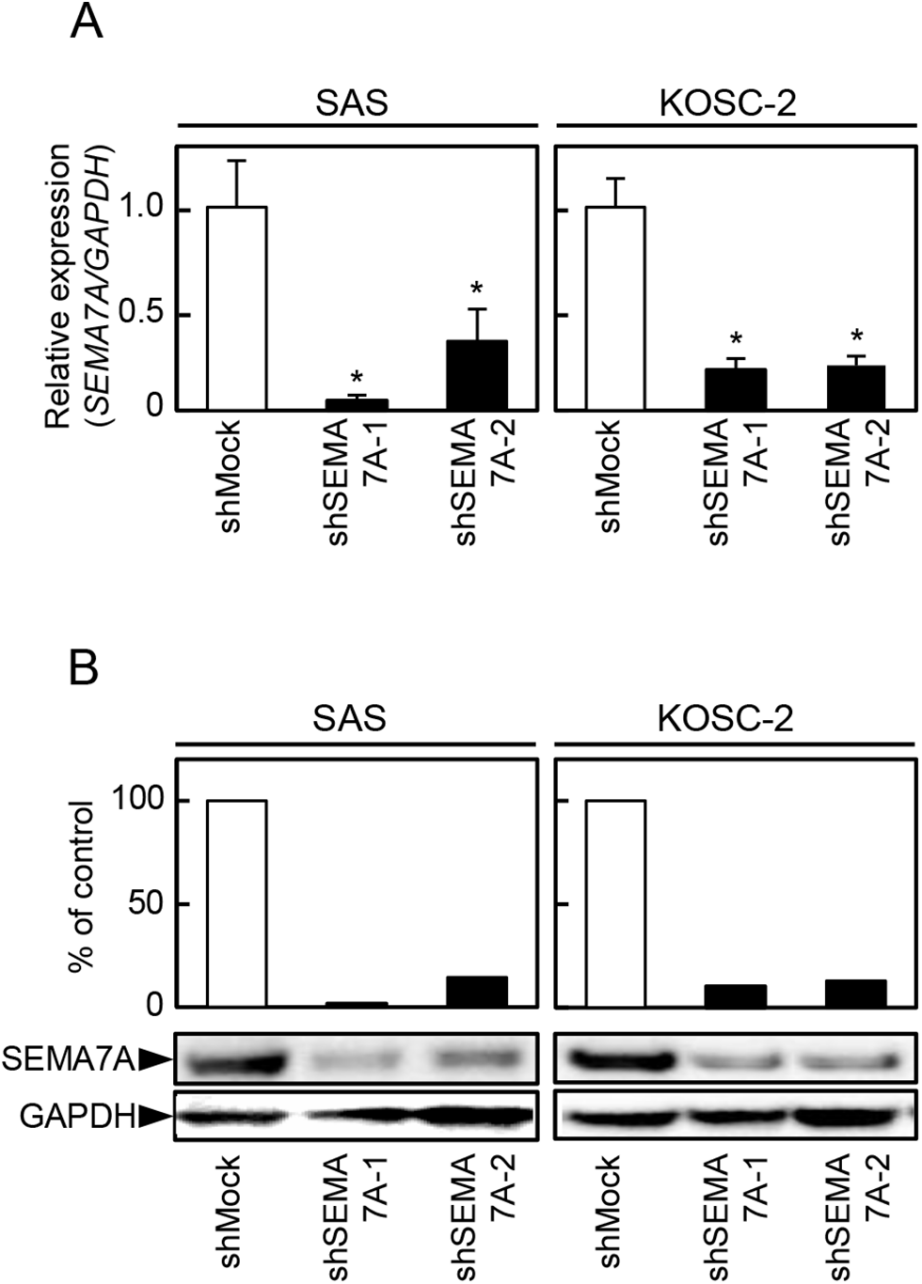
Establishment of SEMA7A knockdown cells. (**A**) Expression of *SEMA7A* mRNA in shMock and shSEMA7A cells (SAS and KOSC-2-derived transfectants). *SEMA7A* mRNA expression in shSEMA7A cells is significantly (^✽^P<0.05, Student’s t-test) lower than in the shMock cells. (**B**) Immunoblotting analysis shows that the SEMA7A protein levels in the shSEMA7A cells also are decreased markedly compared with the shMock cells.

Since frequent up-regulation of SEMA7A was observed in OSCC-derived cells (Fig 1), we transfected SEMA7A shRNA or shMock in OSCC-derived cells (SAS and KOSC-2). To investigate SEMA7A mRNA and protein expressions in shSEMA7A cells, qRT-PCR and immunoblotting analyses were performed (Fig 2A and 2B, respectively). The *SEMA7A* mRNA expression in shSEMA7A cells was significantly (P<0.05) lower than in shMock cells (Fig 2A). The SEMA7A protein level in the shSEMA7A cells also was decreased compared with shMock cells (Fig 2B).

### Functional analyses of SEMA7A knockdown cells

**Fig 3:**
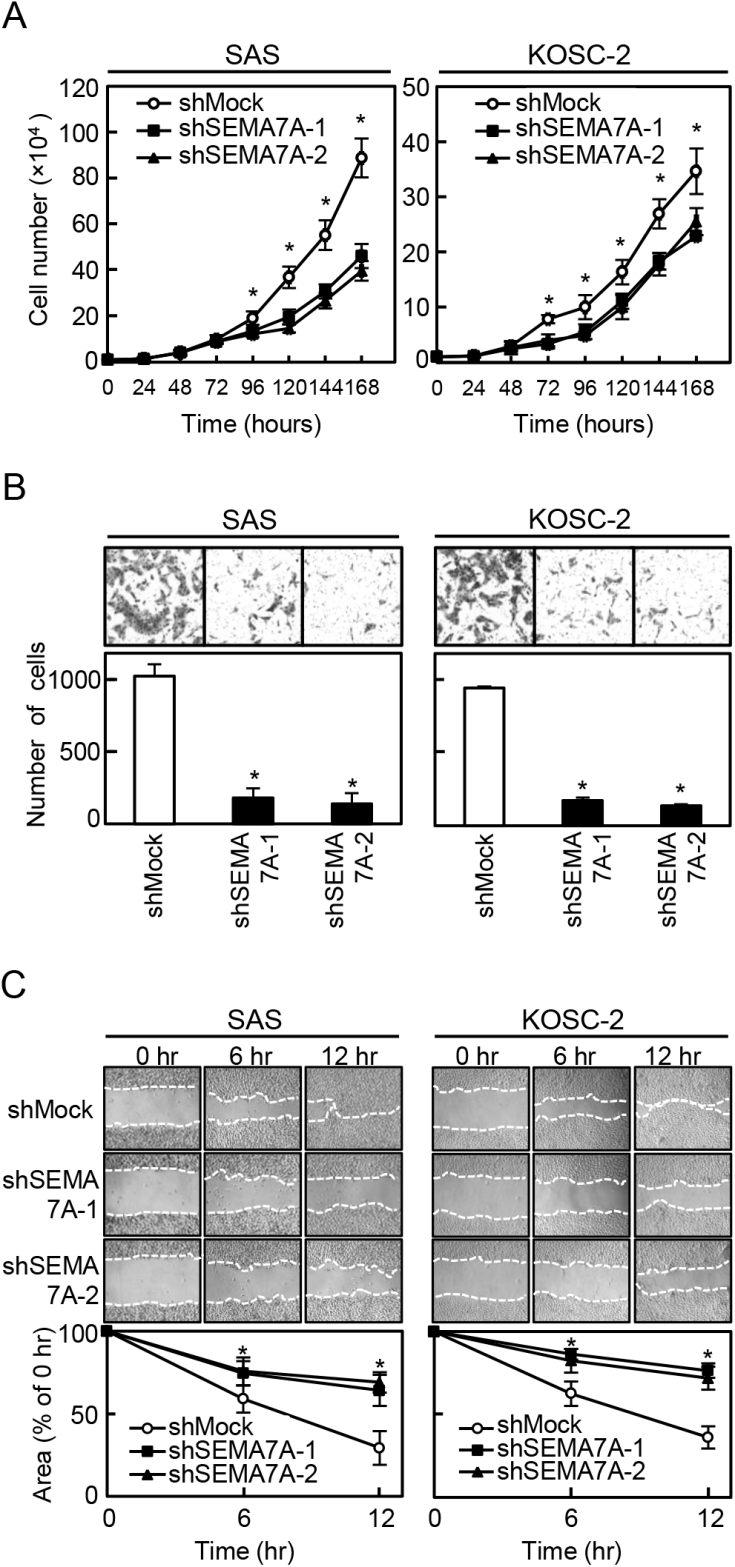
Functional analyses of SEMA7A knockdown cells. (**A**) Cellular proliferation assay of shMock and shSEMA7A cells (SAS and KOSC-2-derived transfectants). To determine the effect of shSEMA7A on cellular proliferation, shMock and shSEMA7A cells were seeded in 6-cm dishes at a density of 1×10^4^ viable cells/well. The cellular growth of shSEMA7A cells is inhibited significantly compared with the shMock cells after 4 days (96 hours). The results are expressed as the means ± SEM of values from three assays (^✽^P<0.05, Student’s t-test). (**B**) Invasiveness assay of shMock and shSEMA7A cells (SAS and KOSC-2-derived transfectants). To evaluate the effect of SEMA7A knockdown on invasiveness, we seeded 2.5×10^5^ cells in the serum-free medium of Matrigel^®^-coated Transwell^®^ inserts (8 μm pores) and added serum-supplemented medium in the lower chamber as a chemoattractant. After incubation at 37°C for 48 hours, cells that penetrated through the pores were fixed, stained, and counted using a light microscope at ×100 magnification. The number of shSEMA7A cells penetrating through the pores is decreased significantly (^✽^P<0.05, Student’s t-test) compared with the shMock cells. The mean value was calculated from data obtained from three separate chambers. (**C**) Migration assay of shMock and shSEMA7A cells (SAS and KOSC-2-derived transfectants). To evaluate the effect of SEMA7A knockdown on migration, uniform wounds were made in confluent cultures of the shSEMA7A and shMock cells and the extent of closure was monitored visually every 6 hours for 12 hours. The mean value was calculated from data obtained from three separate chambers. The wound area is decreased significantly (^✽^P<0.05, Student’s t-test) in the culture of shMock cells after 12 hours, whereas a gap remained in the shSEMA7A cells.

To investigate the effect of SEMA7A knockdown on cellular proliferation, we monitored cellular growth for 168 hours. Cellular growth in shSEMA7A cells decreased significantly (P<0.05) compared with the shMock cells (Fig 3A). We also performed cellular invasiveness and migration assays to assess the biologic effects of SEMA7A knockdown cells. In an invasiveness assay, the number of penetrating shSEMA7A cells significantly (P<0.05) decreased compared with the shMock cells (Fig 3B). In the migration assay, when we visually monitored the area of uniform wounds in confluent cell culture, the wounds in the shSEMA7A cells closed significantly (P<0.05) later than those in the shMock cells (Fig 3C). Therefore, shSEMA7A cells showed not only decreased cellular proliferation but also decreased invasiveness and migratory abilities.

### Cell-cycle analysis of shSEMA7A cells

**Fig 4:**
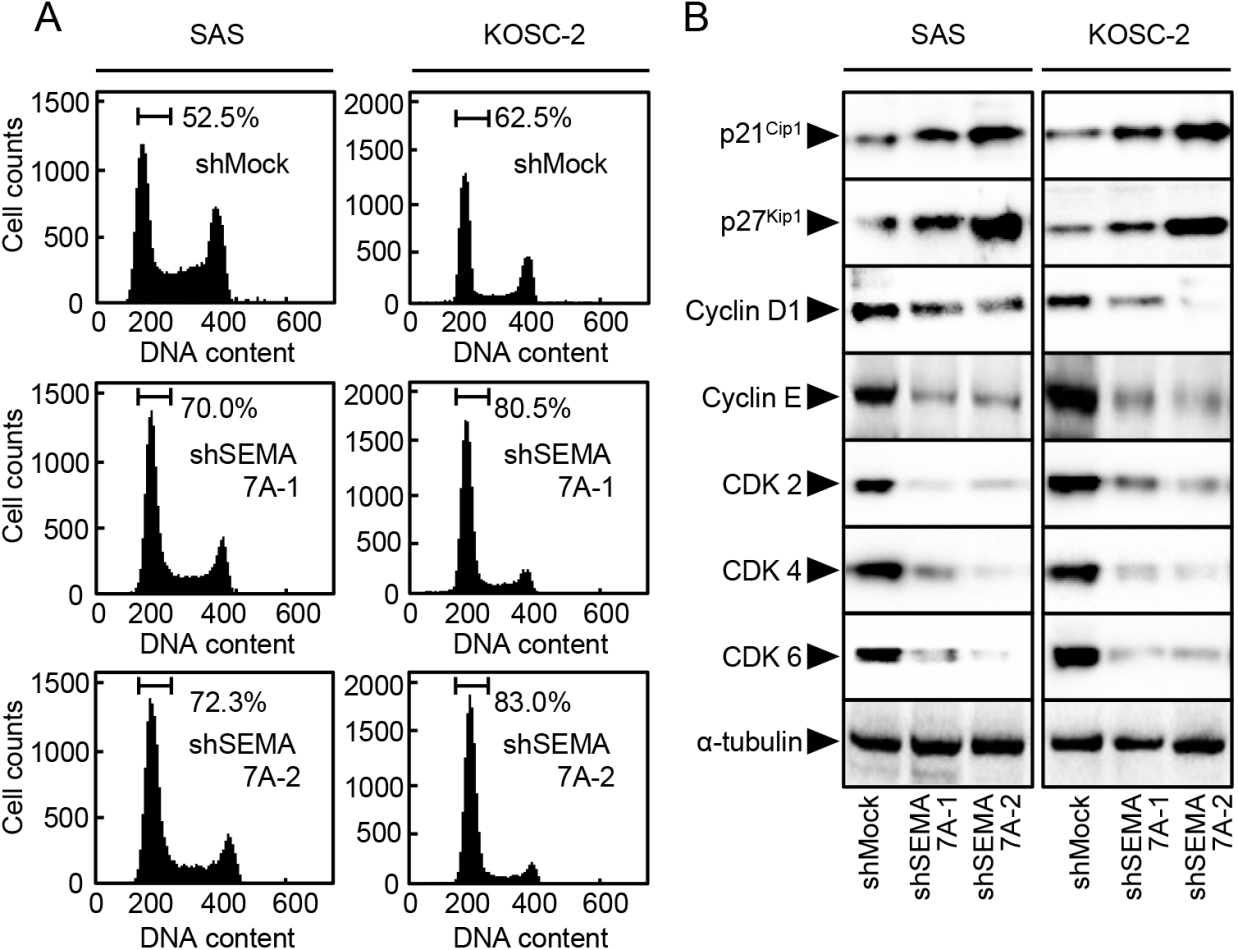
Cell-cycle analysis of SEMA7A knockdown cells. (**A**) Flow cytometric analysis was performed to investigate cell-cycle progression in the shSEMA7A and shMock cells (SAS and KOSC-2-derived transfectants) after synchronization at the G2/M phase to using nocodazole. The percentage of cells at the G1 phase in the shSEMA7A cells is increased markedly compared with the shMock cells. (**B**) Immunobloting analysis shows up-regulation of p21^Cip1^ and p27^Kip1^ and down-regulation of cyclin D1, cyclin E, CDK2, CDK4, and CDK6 in the shSEMA7Acells (SAS and KOSC-2-derived transfectants) compared with the shMock cells.

Since the cellular growth of the SEMA7A knockdown cells decreased (Fig 3A), we investigated the cell-cycle distributions using flow cytometry. The percentage of the cells in the G1 phase in shSEMA7A cells was significantly (P<0.05) higher than that in the shMock cells (Fig 4A).We also assessed the expression levels of the G1 arrest-related proteins, such as CDKIs (p21^Cip1^ and p27^Kip1^), cyclins, and CDKs. As expected, the CDKIs were up-regulated, and cyclin D1, cyclin E, CDK2, CDK4, and CDK6 were down-regulated significantly (P<0.05) in the shSEMA7A cells (Fig 4B). These results indicated that shSEMA7A cells inhibited cellular proliferation by cell-cycle arrest at the G1 phase.

### Reduced secretion of MMPs and expressin of MT1-MMP in SEMA7A knockdown cells

**Fig 5:**
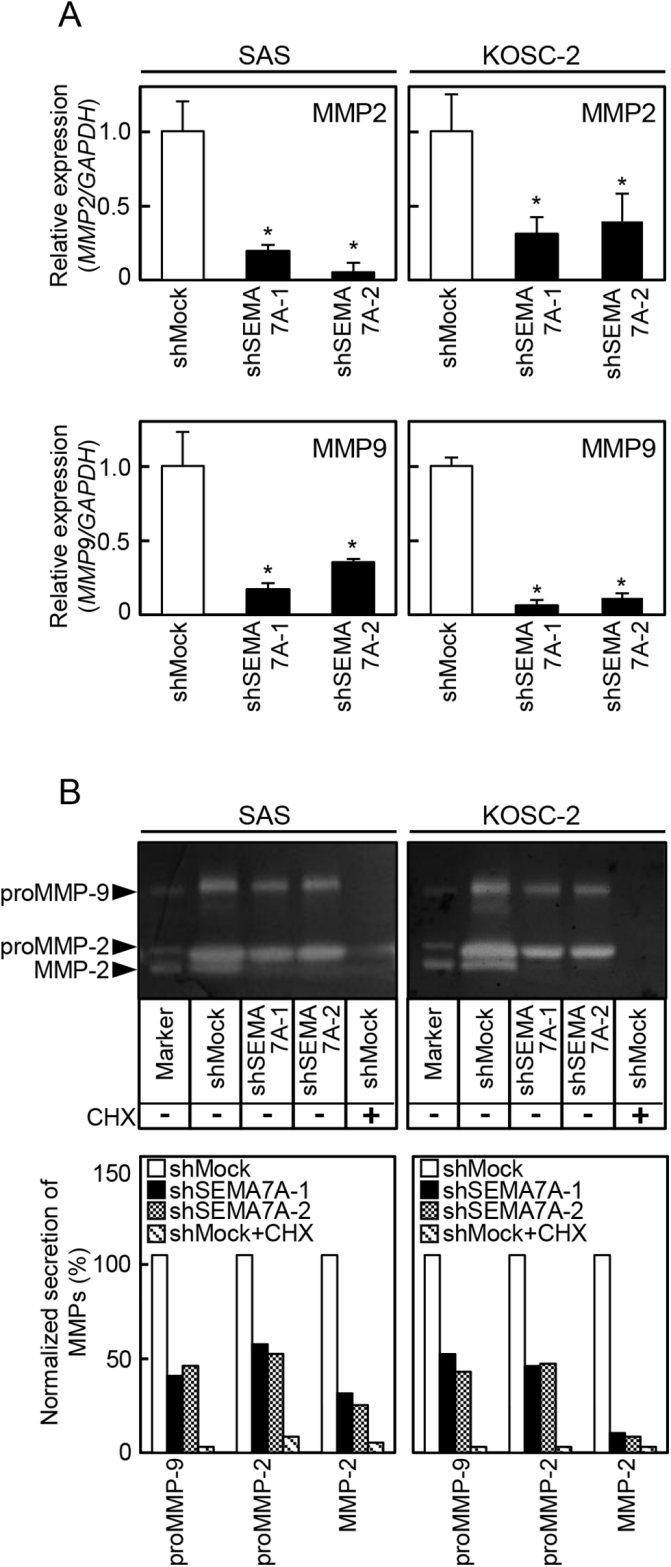
Reduced secretion of MMPs in SEMA7A knockdown cells. (**A**) Expressions of MMP-2 and MMP9 mRNA in shMock and shSEMA7A cells (SAS and KOSC-2-derived transfectants). MMP-2 and MMP9 mRNA expressions in shSEMA7A cells are significantly (✽p<0.05, Student’s t-test) lower than in shMock cells. (B) Secretions of MMP-2, proMMP-2, and proMMP-9 in shMock and shSEMA7A cells were analyzed by gelatin zymography. In the shSEMA7A cells, secretion of MMP2, proMMP-2, and proMMP-9 are clearly decreased compared with shMock cells. In addition, CHX treated shMock cells inhibited the secretion of MMP-2, proMMP-2, and pro-MMP-9. Densitometric MMP-2, proMMP-2, and proMMP-9 levels are normalized to shMock levels.

**Fig 6:**
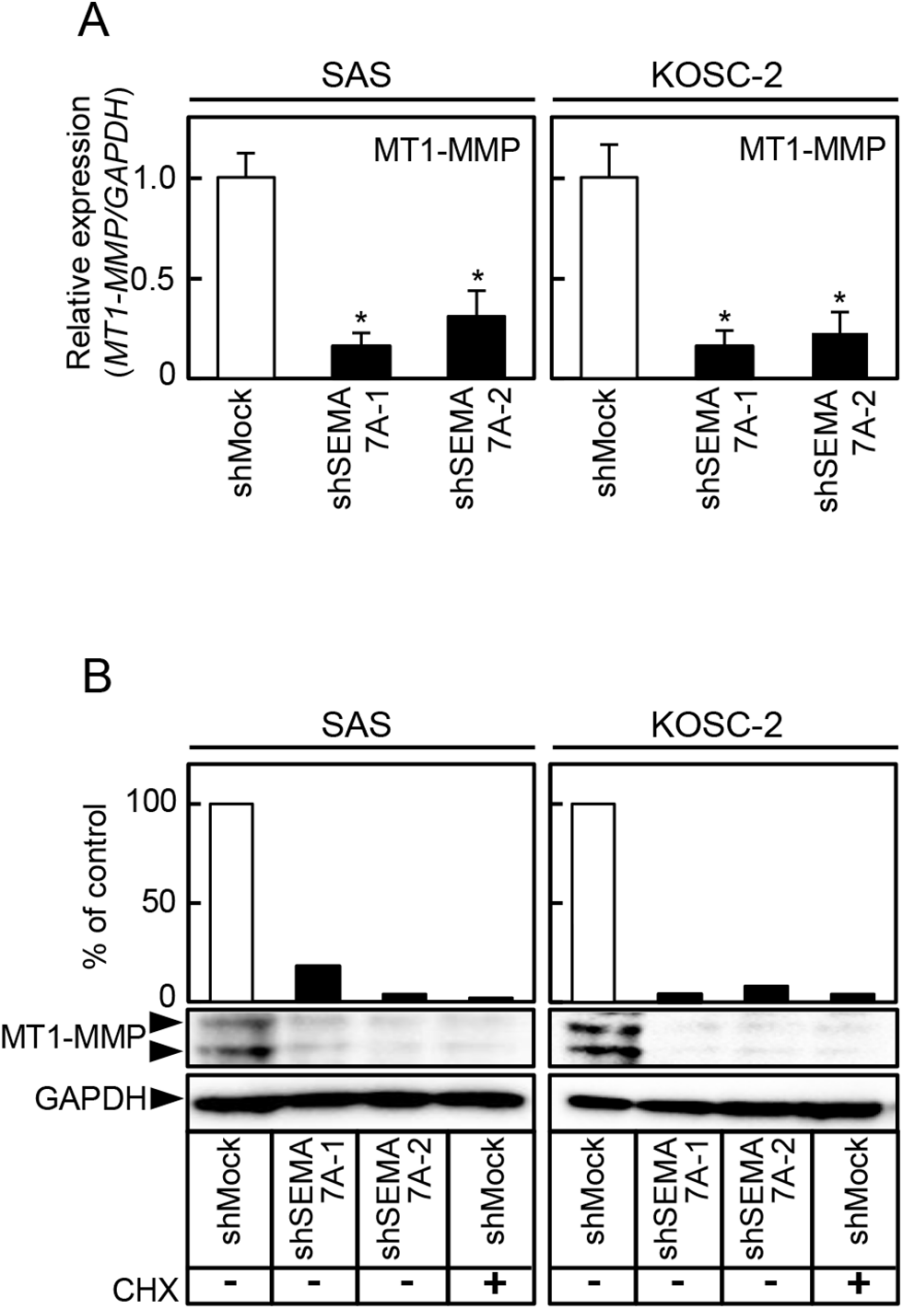
Reduced expression of MT1-MMP in SEMA7A knockdown cells. (**A**) Expression of *MT1-MMP* mRNA in shMock and shSEMA7A cells (SAS and KOSC-2-derived transfectants). *MT1-MMP* mRNA expression in shSEMA7A cells are significantly (^✽^P<0.05, Student’s t-test) lower than in shMock cells. (**B**) Immunoblotting analysis shows that the MT1-MMP protein levels in the shSEMA7A cells are also decreased markedly compared with the shMock cells. CHX treated shMock cells also inihibited the expression of MT1-MMP expression. Densitometric MT1-MMP protein data are normalized to the GAPDH protein levels.

Since the invasiveness and migratory abilities in SEMA7A knockdown cells decreased (Fig 3B and 3C), we assessed MMPs-mediated matrix proteolysis by qRT-PCR and a MMP zymography assay. *MMP2* and *MMP9* mRNA expressions in the shSEMA7A cells were significantly (p<0.05) lower than in the shMock cells (Fig 5A). In the shSEMA7A cells, secretion of MMP2, proMMP-2, and proMMP-9 clearly decreased compared with shMock cells. CHX treated shMock cells inhibited the secretion of MMP-2, proMMP-2, and pro-MMP-9. The levels of secreted MMP were represented as the normalized secretion index, which was calculated as the percentage of secreted MMP relative to that of the shMock cells (Fig 5B). We also assessed MT1-MMP by qRT-PCR and immunoblotting analyses (Fig 6A and 6B, respectively). The *MT1-MMP* mRNA expression in shSEMA7A cells was significantly (P<0.05) lower than in shMock cells (Fig 6A). The MT1-MMP protein level in the shSEMA7A cells also was decreased compared with shMock cells (Fig 6B). CHX treated shMock cells also inihibited the expression of MT1-MMP expression. These findings strongly suggested that SEMA7A was an essential molecule for MMPs’ secretion and expression.

### Inactivation of the ERK1/2 and AKT pathways in SEMA7A knockdown cells

**Fig 7:**
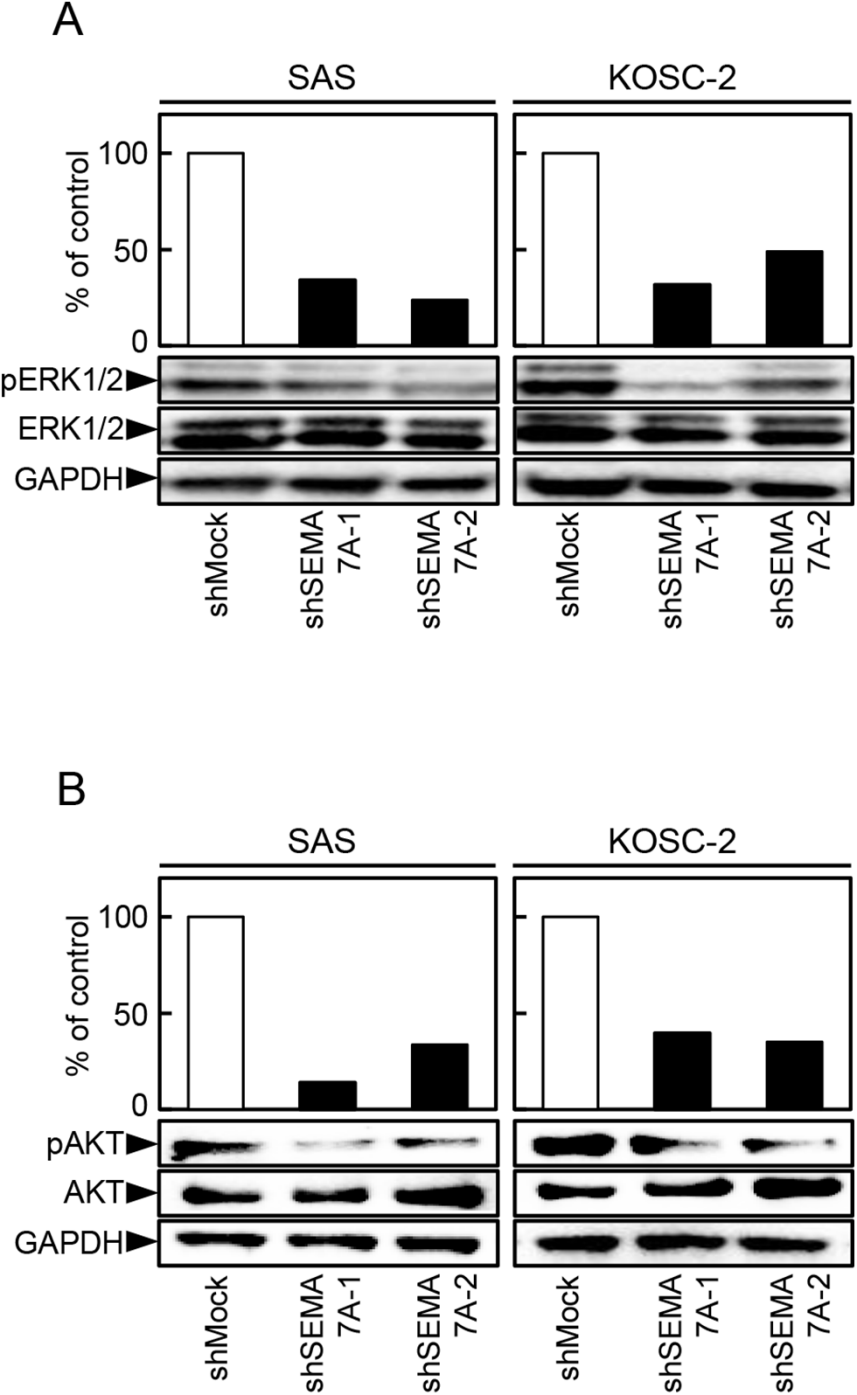
Inactivation of the ERK1/2 and AKT pathways in SEMA7A knockdown cells. (**A, B**) Immunoblotting analysis shows that SEMA7A knockdown results in decreased levels of pERK1/2 and pAKT compared with the shMock cells (SAS and KOSC-2-derived transfectants). Densitometric pERK1/2, ERK1/2, pAKT, and AKT protein data are normalized to GAPDH protein levels.

We assessed the phosphorylation levels of ERK1/2 and AKT in shSEMA7A by immunoblotting analysis. The levels of pERK1/2 and pAKT protein decreased significantly (p<0.05) in the shSEMA7A cells compared with shMock cells (Fig 7A and 7B). These results suggested that the ERK1/2 and AKT signaling pathways were attenuated frequently in the shSEMA7A cells.

### SEMA7A expression in SEMA7A knockdown cells after TGF-β_1_ treatment

**Fig 8:**
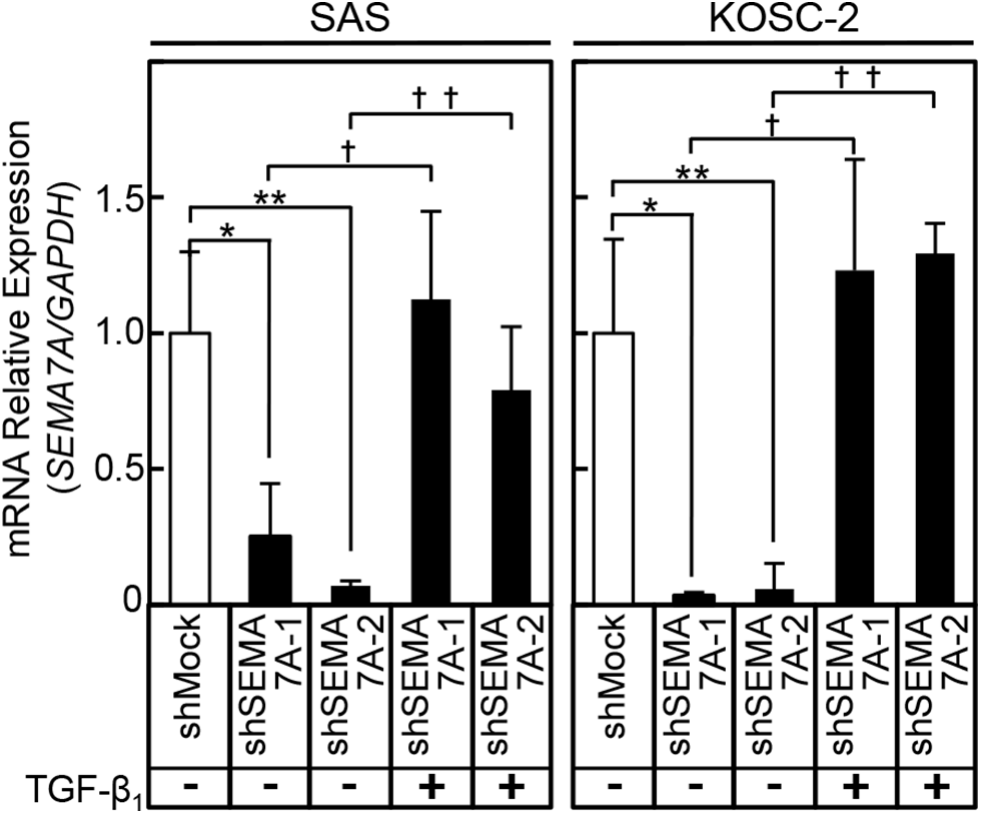
SEMA7A expression in SEMA7A knockdown cells after TGF-β_1_ treatment. The mRNA levels of *SEMA7A* in TGF-β_1_ incubated shSEMA7A cells (SAS and KOSC-2) are increased significantly (SAS, *P = 0.0022, **P = 0.0021, †P = 0.0001, ††P = 0.0005, Student’s t-test) (KOSC-2, *P = 0.0003, **P = 0.0003, †P = 0.0029, ††P = 0.0045, Student’s t-test) compared with that in TGF-β_1_ unincubated shSEMA7A cells.

To investigate the effect of TGF-β_1_ on SEMA7A expression, we treated the transfectants with TGF-β_1_. The *SEMA7A* mRNA in the TGF-β_1_ treated shSEMA7A cells were significantly up-regulated compared with TGF-β_1_ non-treated shSEMA7A cells (Fig 8).

### Phosphorylation levels of ERK1/2 and AKT in SEMA7A knockdown cells with/without TGF-β_1_

**Fig 9:**
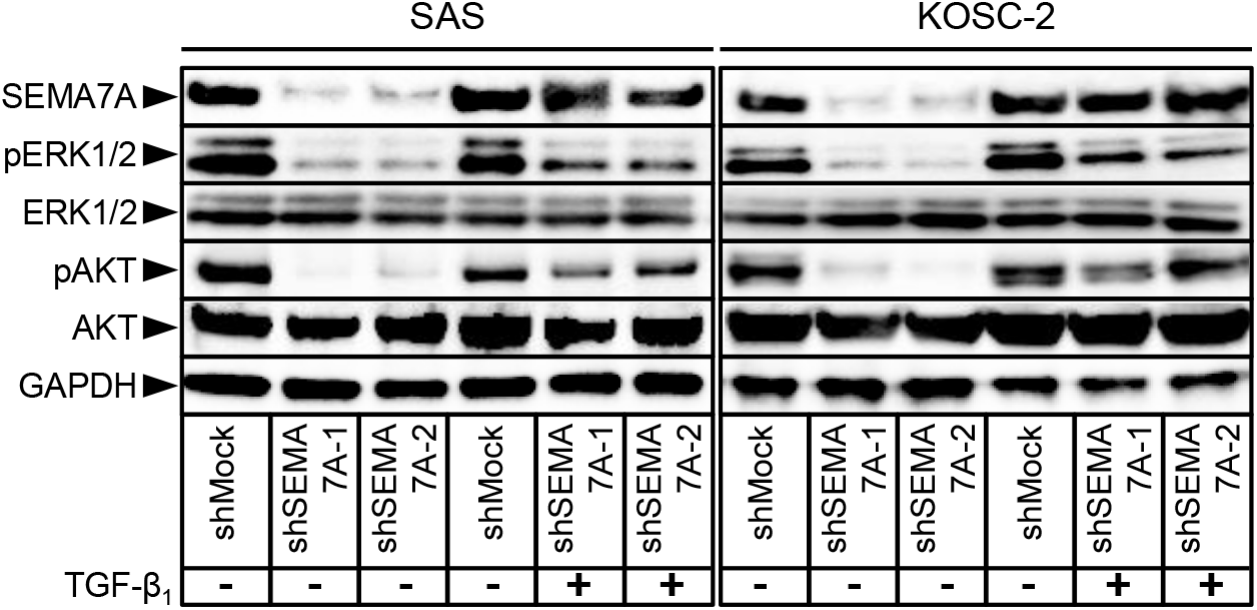
Phosphorylation levels of ERK1/2 and AKT in SEMA7A knockdown cells with/without TGF-β_1_. Immunoblot analysis of the phosphorylation levels of SEMA7A, ERK1/2, and AKT. SEMA7A knockdown results in decreased levels of pERK1/2 and pAKT compared with the shMock cells (SAS and KOSC-2-derived transfectants). TGF-β_1_ treated shSEMA7A cells show increased SEMA7A level compared with TGF-β_1_ non-treated shSEMA7A cells. The pERK1/2 and pAKT level also show increased in TGF-β_1_ treated shSEMA7A cells.

We assessed the phosphorylation levels of ERK1/2 and AKT as well as SEMA7A expression in SEMA7A knockdown cells with/without TGF-β_1_ by immunoblot analysis. The SEMA7A protein level in the TGF-β_1_ treated shSEMA7A cells was increased compared with the non-treated shSEMA7A cells. In addition, TGF-β_1_ treated shSEMA7A cells showed increased pERK and pAKT levels compared with non-treated shSEMA7A cells (Fig 9).

### Evaluation of SEMA7A expression in primary OSCCs

**Fig 10:**
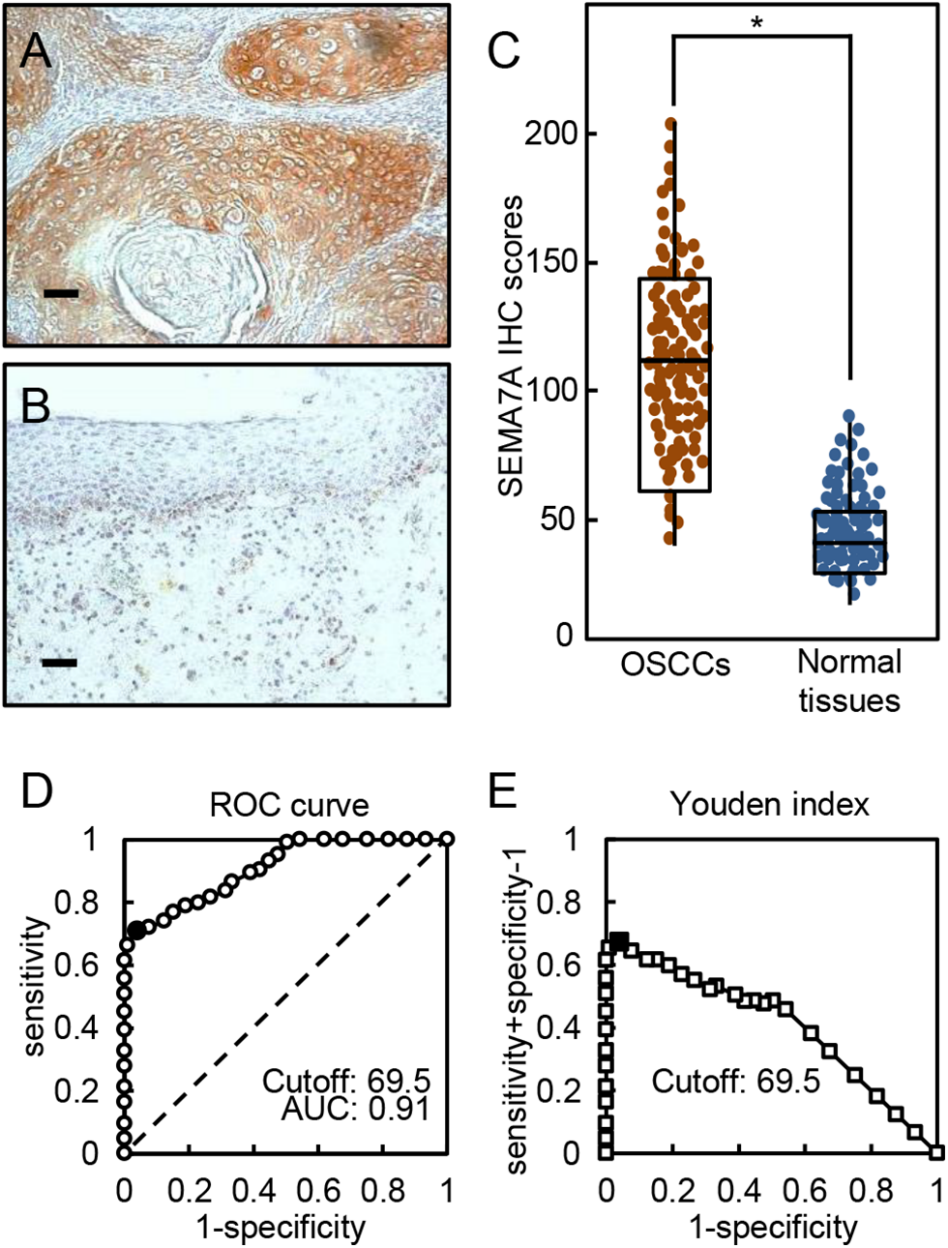
Evaluation of SEMA7A expression in primary OSCCs. (**A, B**) Representative sq-IHC results of SEMA7A in primary OSCCs and normal oral tissues. Original magnification, ×200. Scale bars, 50 μm. (**C**) The status of SEMA7A protein expression in primary OSCCs (n = 150) and normal counterparts based on the sq-IHC scoring system. The SEMA7A sq-IHC scores for OSCCs and normal oral tissues range from 36.7 to 203.8 (median, 109.3) and 13.5 to 87.5 (median, 37.9), respectively. SEMA7A protein expression levels in OSCCs are significantly higher than in normal oral tissues (*P<0.05, Student’s t-test). (**D**) ROC curve analysis revealed that the optimal cutoff point is 69.5 (AUC, 0.91; 95% CI, 0.8749–0.9378; P<0.05). (**E**) Youden index analysis shows that the optimal cutoff point is 69.5 (sensitivity, 70.5%; specificity, 96.7%, P<0.05).

To investigate the expression status of SEMA7A in primary OSCCs and the relation to the clinicopathological characteristics, we analyzed the SEMA7A protein expression in primary OSCCs from 150 patients using a sq-IHC scoring system. The SEMA7A protein expression of primary OSCCs was significantly (P<0.05) higher than in normal tissues (Fig 10A–10C). The SEMA7A sq-IHC scores in OSCCs and adjacent normal oral tissues ranged from 36.7 to 203.8 (median, 109.3) and 13.5 to 87.5 (median, 37.9), respectively (Fig 10C). To determine an optimal cutoff point of the identified sq-IHC scores, we used ROC curve analysis and the Youden index. The ROC curve analysis area under the curve (AUC) was 0.91 (95% confidence interval [CI], 0.8749–0.9378, P<0.05) and Youden index (sensitivity, 70.5%; specificity, 96.7%, P<0.05) showed that the cutoff value was 69.5 (Fig 10D and 10E). The correlations between the clinicopathological characteristics of the patients with OSCC and the status of the SEMA7A protein expression are shown in Table 1. Among the clinical classifications, the SEMA7A-positive OSCCs were correlated significantly (P<0.05) with larger tumors, frequent regional lymph node metastasis, and advanced clinical stages. The 5-year survival rates in the SEMA7A-positive OSCCs (n = 106) and the SEMA7A-negative OSCCs (n = 44) were 76.3% and 85.3%, respectively. The survival rates in the SEMA7A-positive group were lower than in the SEMA7A-negative group, but the difference did not reach significance (P = 0.27) (S1 Fig).

**Table 1:** Correlation between SEMA7A expression and clinical classification in OSCCs.

Clinical classification		Results of immunostaining		
		No. of patients		
	Total	SEMA7A-negative	SEMA7A-positive	p value
**Age at surgery (years)**				
**<60**	**46**	**16**	**30**	**0.6420** ^c^
**≧60, <70**	**47**	**7**	**40**	
**≧70**	**57**	**21**	**36**	
**Gender**				
**Male**	**101**	**29**	**72**	**0.4770** ^b^
**Female**	**49**	**15**	**34**	
**T-primary tumor**				
**T1**	**28**	**13**	**15**	**0.0254** ^c^ ^*^
**T2**	**57**	**17**	**40**	
**T3**	**27**	**6**	**21**	
**T4**	**38**	**8**	**30**	
**N-regional lymph node**				
**Negative**	**93**	**38**	**55**	**0.0002** ^b^ ^*^
**Positive**	**57**	**6**	**51**	
**Stage**				
**I**	**26**	**11**	**15**	**0.0006** ^c^ ^*^
**II**	**43**	**18**	**25**	
**III**	**25**	**7**	**18**	
**IV**	**56**	**8**	**48**	
**Vascular invasion**				
**Negative**	**110**	**35**	**75**	**0.1833** ^b^
**Positive**	**40**	**9**	**31**	
**Histopathologic type**				
**Well**	**99**	**28**	**71**	**0.9762** ^c^
**Moderately**	**42**	**16**	**26**	
**Poorly**	**9**	**0**	**9**	
**Tumoral site**				
**Gingiva**	**39**	**11**	**28**	**0.5851** ^a^
**Tongue**	**89**	**29**	**60**	
**Buccal mucosa**	**8**	**1**	**7**	
**Oral floor**	**11**	**3**	**8**	
**Soft palate**	**3**	**0**	**3**	

Asterisks indicate significance.

^**a**^ **χ** ^**2**^ **test.**

^**b**^ **Fisher’s exact test.**

^**c**^ **Mann-Whitney U test.**

## Discussion

The current study found that SEMA7A was overexpressed frequently in all OSCC-derived cell lines (Fig 1) and that SEMA7A knockdown cells decreased not only cellular proliferation but also invasiveness and migration via cell-cycle arrest at the G1/S phase and inhibited secretion and expression of MMPs through the ERK1/2 and AKT pathways (Figs 3–9). Interestingly, SEMA7A-positive OSCCs were correlated significantly with tumoral size, regional lymph node metastasis, and clinical stages (Fig 10, Table 1).

SEMA7A participates as a tumoral suppressor protein in melanoma progression [34]. In contrast, recent studies have reported that SEMA7A was overexpressed in breast cancer, melanoma, and glioblastoma and may contribute to tumoral cell migration and tissue remodeling in the tumoral microenvironment [2, 35, 36]. Therefore, SEMA7A plays pivotal roles in tumoral progression and metastasis of several types of cancers.

In addition to mediating cellular adhesion, integrins make transmembrane connections to the cytoskeleton and activate many intracellular signaling pathways, tumoral invasiveness, metastasis, and angiogenesis [37–40]. Since SEMA7A contains an integrin-binding motif, RGD28 (amino acids 267–269) [41], inactivation of ERK1/2 and AKT in our SEMA7A knockdown cells may result from SEMA7A-β1-integrin ligation. ERK1/2 and AKT are central mediator for numerous downstream molecules that regulate the Cip/Kip family (p21^Cip1^ and p27^Kip1^) negatively and CDK complexes positively [42, 43]. The Cip/Kip family is implicated in the negative regulation of the cell-cycle progression from the G1 to S phase by modulating CDK activity [44–48]. The activated ERK1/2 and AKT pathways also are related to the invasive phenotype of tumoral cells via up-regulation of MMPs, which proteolytically degrade various components of the extracellular matrix [49]. Of them, MMP-2 and MMP-9 have the unique ability to degrade type IV collagen, a major component of the basement membrane. MT1-MMP is a membrane-tethered MMP that has been shown to facilitate invasion of tumor cells through interstitial collagen by accomplishing proteolysis type I collagen [23, 50]. Accordingly, elevated expressions of MMP-2, MMP-9, and MT1-MMP are associated with increased metastatic potential in many tumoral cells, and inhibition of MMP activities results in reduced tumoral invasiveness and metastasis [51, 52]. In addition to our in vitro data from SEMA7A knockdown models in previous reports, the patients with SEMA7A-positive OSCC tended to have increased tumoral sizes and lymph node metastasis (Table 1).

In conclusion, we explored the biologic role of SEMA7A in human oral cancer in the current study. While further studies are needed to study SEMA7A signaling in the cancer microenvironment, we suggested that overexpression of SEMA7A may directly affect tumoral growth and metastasis in OSCCs and that SEMA7A is a potential biomarker of aggressive tumoral progression and a potential therapeutic target for OSCCs.

## Supporting Information

S1 Fig Five-year survival rate. The SEMA7A expression level is not correlated significantly (P = 0.27, log-rank test) with 5-year survival. The 5-year survival rates in the SEMA7A-positive OSCCs (n = 106) and the SEMA7A-negative OSCCs (n = 44) are 76.3% and 85.3%, respectively.
(TIF)
