# Supplementary figures and images for: Semaphorin7A Promotion of Tumoral Growth and Metastasis in Human Oral Cancer by Regulation of G1 Cell Cycle and Matrix Metalloproteases: Possible Contribution to Tumoral Angiogenesis
Source: PLoS One. 2015 Sep 17;10(9):e0137923. doi: 10.1371/journal.pone.0137923 (PMC4574527; doi:10.1371/journal.pone.0137923)

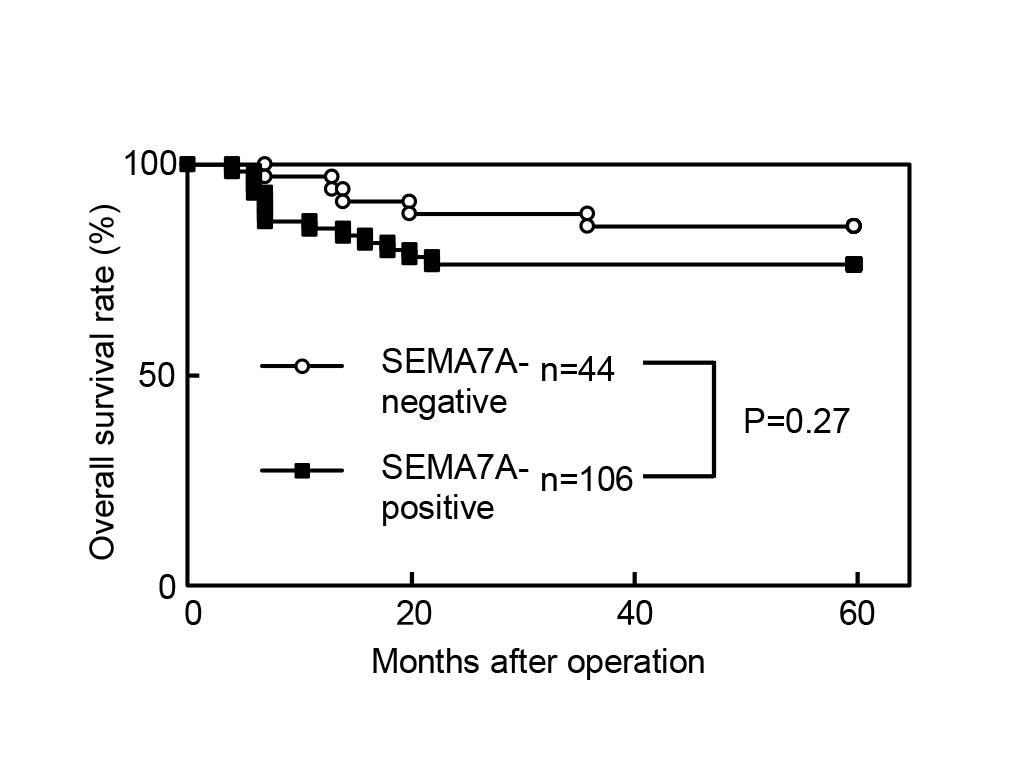

Supplement: S1 Fig — The SEMA7A expression level is not correlated significantly (P = 0.27, log-rank test) with 5-year survival. The 5-year survival rates in the SEMA7A-positive OSCCs (n = 106) and the SEMA7A-negative OSCCs (n = 44) are 76.3% and 85.3%, respectively. (TIF) [file pone.0137923.s001.tif]
